# Morphological Analysis of CDC2 and Glycogen Synthase Kinase 3**β** Phosphorylation as Markers of G2 → M Transition in Glioma

**DOI:** 10.4061/2011/216086

**Published:** 2011-05-24

**Authors:** José Javier Otero, Tarik Tihan

**Affiliations:** Division of Neuropathology, Department of Pathology, University of California, San Francisco, 505 Parnassus Avenue, Moffit-Long Hospital, San Francisco, CA 94143, USA

## Abstract

G2 → M transition is a strategic target for glioma chemotherapy. Key players in G2 → M transition include CDC2 and glycogen synthase kinase 3**β** (GSK3**β**), which are highly regulated by posttranslational phosphorylation. This report is a morphological analysis of CDC2 and GSK3**β** phosphorylation using immunohistochemistry in gliomas with different biological properties. GBM showed a 2.8-fold and 5.6-fold increase in number of cells positive for pThr161CDC2 and a 4.2- and 6.9-fold increase in number of cells positive for pTyr15CDC2 relative to oligodendroglioma and ependymoma, respectively. Elevated labeling for inhibited phospho-CDC2 (pTyr15CDC) correlates with elevated levels of phosphorylated glycogen synthase kinase 3**β** (GSK3**β**). 71% of the GBM cases showed intermediate to high intensity staining for pSer9SGK3**β** 53% of oligodendroglioma, and 73% of ependymoma showed low intensity staining. CDC2 gene amplification correlates with increased survival in glioblastoma multiforme (GBM) and astrocytoma WHO grades II-III, but not in oligodendroglioma WHO grades II-III.

## 1. Introduction

Cell cycle progression is partly regulated by a group of proteins whose expression is cyclical during the cell cycle. These proteins, known as cyclins, exert their function on the cell cycle partly through-regulating the activity of their binding partners, the cyclin dependent kinases (CDKs)/cell division control (CDC) proteins [1]. Cyclin/CDC complexes, in concert with other proteins, control the cell cycle by regulation of multiple cell cycle checkpoints. Although many of the molecular pathways activated in gliomas have been implicated in the G1 → S phase transition of the cell cycle [2], the role of other cell cycle checkpoints is less clear. Furthermore, temozolomide- (TMZ-) induced cell cycle arrest occurs at the G2 → M transition in glioma cell lines [3]. Repair of TMZ-induced DNA damage is critical for TMZ toxicity, and thus the G2 → M transition is a target for chemotherapy. A central player in the G2 → M phase transition is CDC2 (also known as CDK1) [4]. CDC2 is overexpressed in gliomas, and inhibition of CDC2 expression by transfection of small interfering RNA targeted to CDC2 inhibits glioma growth *in vivo *and *in vitro* [5]. 

**Figure 1:**
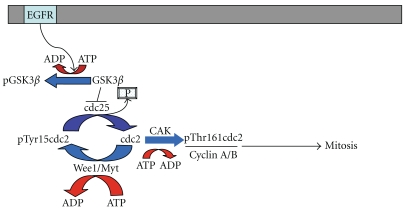
CDC2 pathway is regulated principally by post-translational modification. CDC2 phosphorylation at Tyr15 by Wee1/Myt leads to inactivation of CDC2 and is reversed by the dephosphorylation activity of CDC25A. CDC2 activation is mediated through phosphorylation by cyclin activating kinase (CAK/CDK7) at Threonine 161. Once phosphorylated, CDC2 may promote mitosis by interaction with Cyclins A and B. GSK3*β* is a target of EGFR and may inhibit CDC25's phosphorylation activity, indirectly leading to the inactivation of CDC2. Pathway is adapted from KEGG pathways hsa04110 and has 04115.

CDC2 associates with cyclin-B and cyclin-A. This complex can be either positively or negatively regulated by the state of CDC2 phosphorylation. A model of CDC2 activity is shown in Figure 1. Phosphorylation of a conserved threonine (Thr161) in the T-loop of CDC2 by the CDK Activating Kinase (CAK, also known as CDK7) is required for activation of the cyclin-B/CDC2 complex [4]. Conversely, phosphorylation of CDC2 at threonine 14 (Thr14) and tyrosine 15 (Tyr15) by the Wee1/Mik1 family of protein kinases inhibits the cyclin-B/CDC2 complex [6, 7]. Adding to this complexity, Kang and colleagues demonstrated that CDC25, a promitotic phosphatase that dephosphorylates CDC2 at Tyr15 [8], is targeted for ubiquitin-mediated proteolysis by GSK3*β*- mediated phosphorylation, and that accumulation of CDC25 is highly associated with GSK3*β* inactivation in human tumors [8]. GSK3*β*, a target of the EGF signaling pathway [9], is an ubiquitous kinase with various functions, including but not limited to, initiation of *β*-catenin [10] and cyclin D1 proteolysis [11] and regulation of cell cycle arrest [12]. These and other findings suggest that GSK3*β* may act as a tumor suppressor protein in these setting. Prior studies have shown both CDC2 and GSK3*β* to regulate growth and invasion of cell lines derived from GBM [5, 13]. Evaluation of CDC2 and GSK3*β* activation states in infiltrative primary glial tumors of other lineages has not been thoroughly evaluated. As these proteins' activities are highly regulated through post-translational phosphorylation, a morphological evaluation of their activation states using immunohistochemistry to phospho-specific forms of CDC2 and GSK3*β* was performed.

## 2. Materials and Methods

### 2.1. Patient Demographics and Tissue Samples

 In order to analyze multiple patients simultaneously, tissue arrays composed of glial tumors were generated. The patients had been diagnosed and/or treated at UCSF between 1990–2004. Diagnostic guidelines from the 2007 WHO grading system for CNS tumors were used in this study. Tissue arrays composed of neurosurgical samples from 45 patients with GBM, 37 patients with oligodendroglioma (20 patients with WHO grade II; 17 patients with WHO grade III), and 20 patients with ependymoma were examined. Insufficient numbers of astrocytoma grades II-III were available to perform similar analyses. All GBM cases were newly diagnosed. In the GBM group, 30% (15 of 45 patients) were female, mean age was 54 years, and the median age was 57 years (mode was 40 years). In the WHO II oligodendroglioma group, 20% (6 of 20 patients) were female, mean age was 39 years, and the median age was 41 years (mode was 41 years). In the WHO III oligodendroglioma group, 60% (10 of 17 patients) were female, mean age was 46 years, and the median age was 44 years (mode was 42 years). In the ependymoma group, 70% (14 of 20 patients) were female, mean age was 24 years, and the median age was 19 years (mode was 5 years). The ependymoma group is composed of 9 pediatric patients younger than 15 years and 11 patients greater 15 years. All cases have been reviewed by both authors to confirm the original diagnosis. All oligodendroglioma cases were confirmed by codeletion of 1p19q by FISH using standard procedures. The original hematoxylin and eosin (H&E) stained slides were used to determine tumor cell rich areas for selection of cores from the paraffin-embedded tissue blocks. Tumor cell rich areas were then targeted for extraction, and composite arrays containing “tissue cores,” each measuring 0.1 cm in diameter, were generated using the Beecher Instruments Tissue Arrayer (Beecher Instruments, Inc., Sun Prairie, WI) using the protocol suggested by the manufacturer. A minimum of two cores was obtained from each patient. This approach allows one to compare labeling of different antibodies in “replica” tissue cores simultaneously. The area of each tissue core is 7.85 × 10^−3^ cm^2^. For this study, each high power magnification field was 0.237 mm^2^. Each tissue array slide was composed of one tumor type, and this information was available to the reviewing pathologist. However, each sample had at least another sample from a similar tumor-rich region of the patient sample. The reviewing pathologists were blinded as to the patient ID of the tissue cores. High concordance in results from different tissue cores with the same patient ID was noted.

### 2.2. Optimization of Immunohistochemistry

The following primary antibodies were used in this study: anti-Phospho-Tyr15-CDC2 (Cell Signaling no.4539, Danvers, Mass); anti-Phospho-Thr161-CDC2 (Cell Signaling no.9114, Danvers, MA); anti-Phospho-Ser9-GSK3*β* (pGSK, Cell Signaling no.9336, Danvers, MA); anti-EGF receptor (Oncogene, Cambridge, MA). Immunohistochemical reactions were performed on formalin-fixed, paraffin-embedded tissue using standard procedures. White matter and cortical sections procured from the UCSF autopsy service were used as negative controls. Immunohistochemical optimization was performed by serial titration of antibodies on formalin-fixed, paraffin-embedded tissue sections obtained from the UCSF Pathology Archives. Anti-phospho-Thr161CDC2 (pThr161CDC2) shows optimal staining at 1 : 50 dilution in invasive ductal carcinoma of the breast. Anti-phospho-Tyr15-CDC2 (pTyr15CDC2) shows optimal staining at 1 : 25 dilution in colorectal adenocarcinoma. Anti-phospho-Ser9GSK3*β* ( )pSer9GSK3*β*) shows optimal staining at 1 : 50 in colorectal adenocarcinoma. EGFR, pTyr15CDC2, pThr161CDC2, showed no labeling in normal white matter and cortex. EGFR immunolabelling was detected in the syncytiotrophoblastic cells of placenta, which served as our positive control. Focal positive nuclear labeling of pThr161CDC2 and pTyr15CDC2 was also noted in carcinoma of breast metastatic to brain and in normal lymph nodes.

### 2.3. Quantification of CDC2 Labeling

All immunohistochemical labeling was scored by counting all the cells of interest in three high power magnification fields. Only the cells with similar or higher staining intensity as the control tissue were counted as positive. Occasional positive staining was noted sporadically in endothelial cells. Endothelial cells were not included in the CDC2 quantifications. The total area counted constituted approximately 10% of each tissue core. The CDC2 cell labeling was performed according to previously published counting methods with some modifications [14]. Briefly, cores with no CDC2 labeling are score = 0. Score = 1 shows 1–10 CDC2 positive cells per three high power fields, score = 2 shows 11–20 CDC2 cells per three high power fields, and score = 3 shows 21 or more CDC2 positive cells per three high power fields. To determine the mean CDC2 labeling in the patient, the total number of cells counted were averaged over all of the cores from the same patient. The final patient score was designated as low (scores 0 and 1), intermediate (score 2), or high (score 3). As the tissue cores represent a fraction of the original tissue, a separate category of “negative” was considered inaccurate. Hence, Score = 0 and Score = 1 were grouped together in the low labeling category. Percent positive cells were determined as follows: (#CDC2+ cells/all tumor cells quantified by hematoxylin counterstain)×100.

### 2.4. Quantification of PSerGSK3*β* and EGFR

The scoring method used in evaluation of the pSerGSK3*β* and EGFR immunohistochemical stains was adopted from Atkins et al. [15]. Briefly, tumors with less than 10% labeling in tumor cells were considered negative (score 0). In all others, a minimum of 10% staining of tumor cells were required. Tumors with minimal labeling intensity were scored as LOW (score 1). Intermediate labeling was designated to cores if moderate labeling was detectable in more than 10% of the tumor cells (score 2). A high labeling designation was awarded to cores where more than 10% of the tumor cells displayed strong immunolabeling (score 3). Both authors evaluated the samples with similar results. The labeling score for EGFR and pSer9GSK3*β* is based on immunohistochemical intensity. Different cores from the same patient occasionally showed a variable labeling index, ranging from a low score to a high score. In these patients, the highest labeling score from the various cores is reported. The patient data is illustrated in Table 1. To determine if the labeling indexes of CDC2 results were dependent on cell densities, the percentage labeling in select cases is quantified. In these calculations, the denominator is the total number of tumor cells, and a minimum of 1000 tumor cells were counted per sample.

### 2.5. Statistical Analysis and Survival Data

 Analysis of variance (ANOVA)/Tukey-HSD, Chi-squared test, and heat maps of the Bredel et al. dataset [16] were performed with R v2.12, an open source programming language and statistical framework (http://cran.r-project.org/mirrors.html). Data from the Rembrandt Database [17] were queried for survival data related to expression and copy numbers of CDC2, CDK7/CAK, WEE1, GSK3*β*, Cyclin A2, and Cyclin B1 in patient populations diagnosed with GBM, Astrocytoma WHO grades II-III, and Oligodendroglioma WHO grades II-III. The data from these queries are presented in Figures 8–10 and Tables 2–4. Log ranked *P*-value statistic was calculated by REMBRANDT software on-line (https://caintegrator.nci.nih.gov/rembrandt/registration.do). The query was performed on December 30, 2010.

## 3. Results

### 3.1. Infiltrative Glial Tumors Show Diverse Levels of CDC2 Phosphorylation by Immunohistochemistry

**Figure 2:**
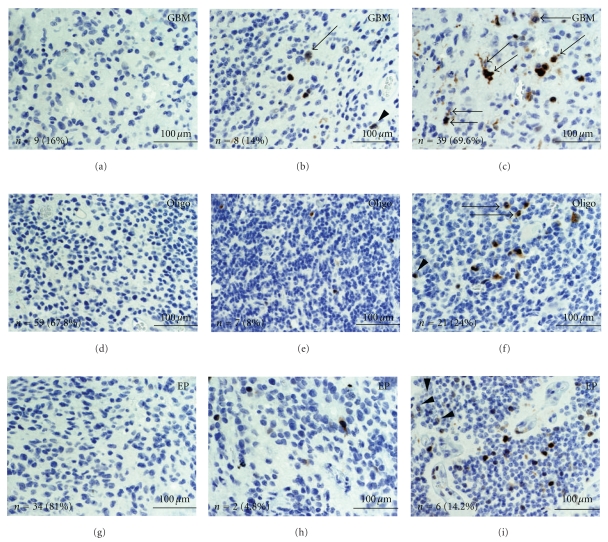
pThr161CDC2 levels in glial tumors. Tissue arrays for GBM (a–c), oligodendroglioma (d–f), and ependymoma (g–i) were stained with anti-pThr161CDC2 antibodies. Groups were separated into low labeling (a, d, and g), intermediate labeling (b, e, and h), and high labeling (c, f, i) and representative photographs are illustrated. Bottom left of each panel shows the total number of cores (*n*) and their proportion of total cores for the low, intermediate, and high labeling group. Arrowheads show nuclear pThr161CDC2 labeling. Arrows show cytoplasmic pThr161CDC2 labeling in mitotic cells. Double arrows show pThr161CDC2 in non-mitotic cells. A greater proportion of cores from the GBM samples were in the high labeling group. (GBM = glioblastoma; Oligo = oligodendroglioma; EP = ependymoma; scale bar in yellow represents 100 *μ*m).

**Figure 4:**
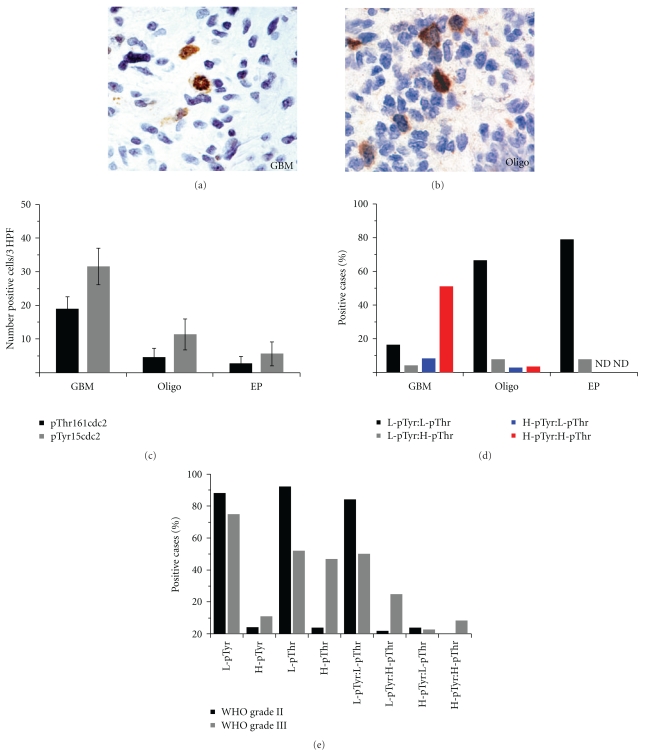
High-grade gliomas show increased phospho-CDC2 levels relative to low-grade gliomas. (a) shows a mitotic figure from a GBM showing strong labeling for pTyr15CDC2; in (b), a mitotic figure from an anaplastic oligodendroglioma (WHO grade III) is shown with strong pTyr15CDC2 labeling. (c) shows the average number of positive cells/3 high power fields of the GBM, oligodendroglioma (Oligo), and ependymoma (EP) arrays (error bars show standard deviation). (d) shows the intracase correlation between pTyr15CDC2 and pThr161CDC2. The *Y*-axis of (d) is the percentage of total cores (i.e., denominator = no. of cores examined for each tumor type). The black bar denotes the percentage of cores where there was both low pTyr15CDC2 and low pThr161CDC2 labeling. The grey bar denotes percentage of cores where there is low pTyr15CDC2 with high pThr161CDC2 labeling. The blue bar denotes percentage of cores where there is high pTyr15CDC2 with low pThr161CDC2 labeling. The red bar denotes cores where there is high pTyr15CDC2 with high pThr161CDC2. Half of the GBM cores showed high pTyr15CDC2 with high pThr161CDC2 labeling, whereas the most common pattern seen in the oligodendroglioma and ependymoma cores was low pTyr15CDC2 with low pThr161CDC2. (e) shows the results of pTyr15CDC2 and pThr161CDC2 labeling observed in oligodendroglioma grade II versus grade III. Oligodendroglioma WHO grade III showed more cores with elevated CDC2 labelling. (pTyr = pTyr15CDC2; pThr = pThr161CDC2; L = low labeling; H = high labeling; ND = none detected).

GBMs showed elevated pThr161CDC2 (activated) labeling relative to oligodendrogliomas and ependymomas (Figure 2 and Table 1). The mean pThr161CDC2 positive cells in GBM was 2.8-fold and 5.6-fold higher than oligodendrogliomas and ependymomas, respectively (Figure 4(c)). Similarly, anaplastic/WHO Grade III oligodendroglioma showed more labeling relative to WHO grade II oligodendroglioma (Figure 4(e)). Mean percent pThr161CDC2 labeling in GBM was 18% (SEM = 5.2), whereas for oligodendroglioma grade II percent labeling was 1.7% (SEM = 0.8) and for ependymoma percent labeling was 2.3% (SEM = 1.5). Of note, all patients with ependymoma showing elevated pThr161CDC2 labeling were found in the pediatric cohort (Table 1). The pThr161CDC2 cell counts from the GBM group were statistically significant from both the oligodendroglioma and ependymoma group (*P* < .05 by Tukey HSD Test). pThr161CDC2 cell counts were not statistically significant between the oligodendroglioma and ependymoma group.

Three distinct labeling patterns are noted in the pThr161CDC2 positive cells. In non-mitotic cells, the predominant labeling pattern was nuclear (Figure 2(f), arrow heads). In mitotic cells, the pThr161CDC2 stain showed cytoplasmic localization (Figures 2(b), 2(c), and 2(f), single arrows). All mitotic cells identified showed pThr161CDC2 labeling in the GBM, oligodendroglioma, and ependymoma samples. However, in 13 of the GBM cores (17.3% of total cores), the pThr161CDC2 labeling showed cytoplasmic localization in *non-mitotic* cells (Figure 2(c), double arrow). Cytoplasmic labeling in non-mitotic cells was not identified in the oligodendroglioma and ependymoma cores. 

 Quantification of the number of positive cells per three high power fields demonstrated elevated pTyr15CDC2 (inhibited) labeling in GBM compared to oligodendrogliomas and ependymomas (Figure 4). pTyr15CDC2 in GBM was 4.2-fold and 6.9-fold-increased relative to oilgodendrogliomas and ependymomas, respectively (Figure 4(c)). These data are also confirmed by quantification of the percent positive cells. In GBM, the percent positive pTyr15CDC2 is 17% (SEM = 4.1), whereas in oligodendroglioma grade II percent positive pTyr15CDC2 is 5.4% (SEM = 2.2) and Ependymoma is 1.7% (SEM = 0.6). For the pTyr15CDC2 cell counts, the cell counts from the GBM group were statistically significant from the oligodendroglioma and ependymoma group (*P* < .05 by Tukey-HSD Test). pTyr15CDC2 counts were not statistically significant between the oligodendroglioma and ependymoma groups.

 The relationship of pTyr15CDC2 to pThr161CDC2 in each individual core was evaluated and the results plotted in Figure 4(d). The majority of oligodendroglioma and the ependymoma cores showed low pTyr15CDC2 labeling with low pThr161CDC2 labeling. In the GBM arrays, half of the cores showed high pTyr15CDC2 labeling with high pThr161CDC2 labeling. The three oligodendroglioma cores showing high pTyr15CDC2 labeling with high pThr161CDC2 labeling were anaplastic/WHO grade III oligodendrogliomas, but this group constituted less than 10% of total anaplastic oligodendrogliomas (Figure 4(e)). Although rare, pTyr15CDC2 positive mitotic was noted in GBM and in one patient with anaplastic/WHO grade III oligodendroglioma (Figures 4(a) and 4(b)). There were no mitotic pTyr15CDC2 positive cells identified in the WHO grade II oligodendroglioma or the ependymoma arrays. Nearly half of the WHO grade III oligodendroglioma cores show elevated pThr161CDC2, whereas in the WHO grade II oligodendroglioma, only 4% show elevated pThr161CDC2 (Figure 4(e)). This is in contrast to pTyr15CDC2 where the percentage of low labeling cores in grade II and grade III oligodendroglioma are 88% and 75%, respectively. There is no statistically significant association between pThr161CDC2 and pTyr15CDC2 labeling with the MIB-1 labeling indexes obtained from the tissue cores (data not shown).

### 3.2. Infiltrative Glial Tumors Show Diverse Levels of Phosphorylated GSK3*β* by Immunohistochemistry

**Figure 5:**
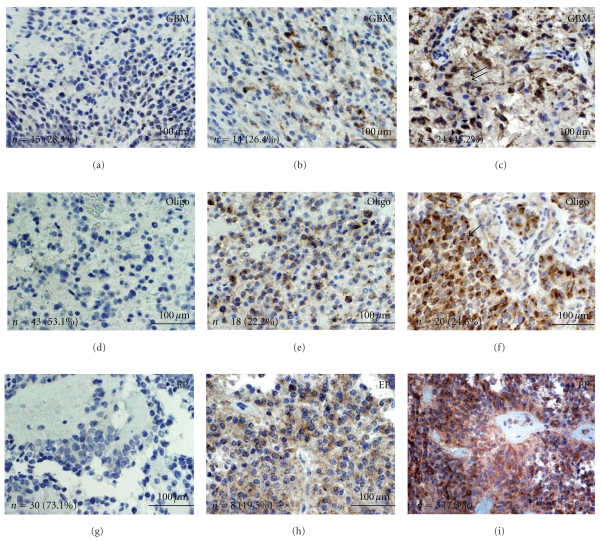
pSer9GSK3*β* labeling in glial tumors. Tissue arrays for GBM (a–c), oligodendroglioma (d–f), and ependymoma (g–h) were stained with pSer9GSK3*β* antibodies. Groups were separated into low labeling (a, d, and g), intermediate labeling (b, e, and h), and strong labeling intensity (c and f) as described in Section 2, and pictures representative of these groups for the three tumors is presented. The number of cores positive and their corresponding percentages are placed in the bottom left of each panel. Perinuclear/cytoplasmic labeling predominates (arrow, (f)), but nuclear (arrowhead, (c)) and labeling in glial processes also was found (double arrows, (c)). (GBM = glioblastoma; Oligo = oligodendroglioma; EP = ependymoma; scale bar in yellow represents 100 *μ*m).

**Figure 6:**
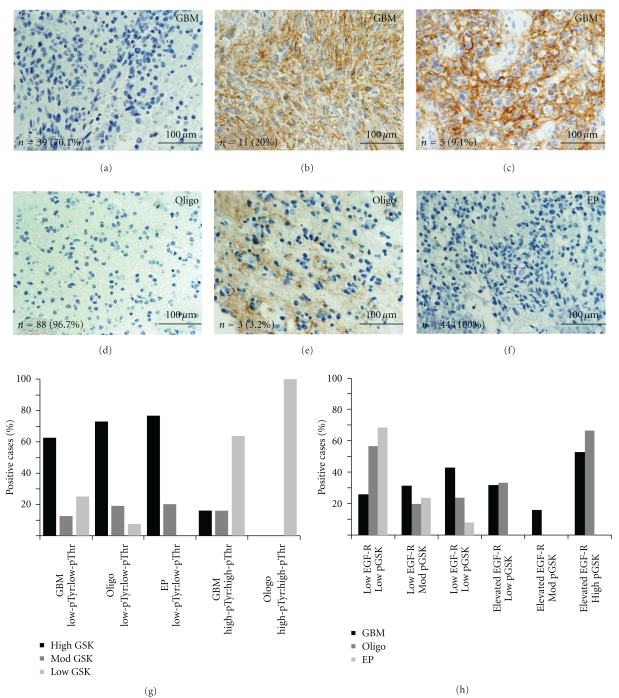
Elevated EGFR labeling correlates with increased p-GSK*β* labeling. Tissue arrays for GBM (a–c), oligodendroglioma (d-e), and ependymoma (f) were stained with EGFR antibodies. Tissue cores showing membrane labeling for EGFR were quantified and pictures representative of these groups for the three tumors is presented. None of the ependymomas showed EGFR immunoreactivity. The number of cores positive and their corresponding percentages are placed in the bottom left of each panel. Correlations between pSer9GSK3*β* levels and phosphorylation state of CDC2 are plotted in (g). Elevated pSer9GSK3*β* levels are highly correlated with elevation of both activated and inhibited CDC2. Correlation between pSer9GSK3*β* levels and EGFR levels are plotted in (h). Most GBM cases show EGFR0020 expression correlated with GSK3*β* phosphorylation.

 Most GBM cores (71% of total cores) showed intermediate to highly elevated levels of pSer9GSK3*β* (Figures 5(a)–5(b), and 5(c)). In contrast, roughly half of the oligodendroglioma (53%) cores and the majority of the ependymoma (73%) showed low pSer9GSK3*β*. Increased grade in the oligodendroglioma and ependymoma groups was correlated with higher pSer9GSK3*β* labeling. 35.7% grade III oligodendroglioma cores showed high pSer9GSK3*β* compared to 20.8% of grade II oligodendroglioma cores. This trend is also observed when comparing individual cases. The predominant labeling pattern observed was cytoplasmic/perinuclear (Figure 5(f), single arrow). However, in 21 of the GBM cores (31.8%), distinct nuclear localization was observed (Figure 5(c), arrowheads). Only one oligodendroglioma core (1.2%) and two ependymoma cores (6.1%) showed nuclear pSer9GSK3*β*. Furthermore, 17 GBM cores (25%) showed pSer9GSK3*β*   labeling in the processes of the tumor cells (Figure 5(c), double arrows), whereas such labeling was present in only two oligodendroglioma cores (2.5%). The presence of pSer9GSK3*β* labeling highly correlates to CDC2 phosphorylation in all three glial tumors studied (Figure 6(g)). The majority of tumors with low pSer9GSK3*β* labeling showed low levels of both pTyr15- and pThr161-CDC2 levels, whereas the majority of oligodendroglioma and the GBM cores with high pSer9GSK3*β* also had high levels of both pTyr15- and pThr161-CDC2 levels (Figure 6(g)). This trend was particularly evident in the GBM and oligodendroglioma samples. 

Correlation between pSer9GSK3*β* levels and EGF receptor overexpression was tested by labeling with anti-EGF receptor antibody (Figure 6). Nearly thirty percent of the GBM cores had either moderately or highly elevated EGF receptor membrane labeling (Figures 6(a)–6(c) and, Table 1), whereas only three oligodendroglioma cores (3.2%) and no ependymoma cores showed EGF receptor expression by immunohistochemistry (Table 1). Although half of the GBM cores with high EGF receptor expression showed elevated pSer9GSK3*β* labeling, roughly 40% of the cores with elevated pSer9GSK3*β* staining were associated with low EGF receptor expression (Figure 6(h)).

### 3.3. G2 → M Checkpoint Protein Transcript Levels Are Changed in GBM Cell Lines after Creating Resistance to the O-6 Methylating Drugs Temozolomide and BCNU

**Figure 7:**
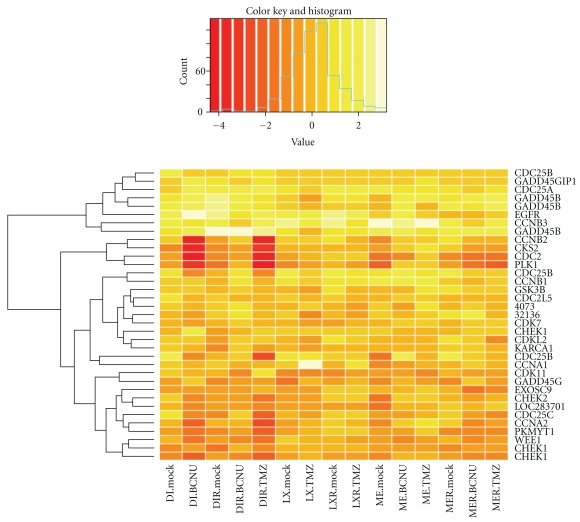
CDC2 interacting proteins and G2 →  M transition regulators change expression levels after TMZ and BCNU treatment. For experimental details, see Bredel et al. [16]. GBM cell lines from three patients (DI, LX, and ME) were derived at initial surgery and then selected *in vitro* for resistance to BCNU or TMZ (DI.mock = no *in vitro* selection in first surgery, ME.TMZ = *in vitro *selection for TMZ from cell line derived from initial surgery, etc.). These patients showed recurrence that was treated surgically, and cell lines from these recurrent/treated tumors were derived (DIR, LXR, MER, R = recurrent). The recurrent cell lines were also selected *in vitro* for resistance to BCNU or TMZ (mock = no *in vitro* selection). Heirarchical clustering of the genes (right) is shown on the left of the heatmap. Gene names are cell division cycle 25A = CDC25A; CDC28 protein kinase regulatory subunit 2: ckshs2 = homolog of Cks1 = p34Cdc28/Cdc2-associated protein = CKS2; Cyclin-dependent kinase 7/CAK = CDK7; cyclin-dependent kinase (CDC2-like) 11 CDK11; epidermal growth factor receptor = EGFR; Cyclin B2 = CCNB2; Cyclin B1 = CCNB1; cyclin B3 = CCNB3; cell division cycle 2-like 5 = CDC2L5; cell division cycle 2 = CDC2; glycogen synthase kinase 3 beta = GSK3*β*; cell division cycle 25C = CDC25C; WEE1 homolog = WEE1; cell division cycle 25A = CDC25A; cell division cycle 25B CDC25B; cell division cycle 25B = CDC25B; membrane-associated tyrosine- and threonine-specific cdc2-inhibitory kinase: Myt1 kinase = PKMYT1; similar to Serine/threonine-protein kinase PLK1 (Polo-like kinase 1) (PLK-1) (serine-threonine protein kinase 13) (STPK13) = 4073 and 32136; Polo-like kinase 1 = PLK1; CHK1 checkpoint homolog = CHEK1; growth arrest and DNA-damage-inducible, gamma interacting protein 1 = GADD45GIP1; growth arrest and DNA-damage-inducible, beta = GADD45B; growth arrest and DNA damage inducible, gamma = GADD45G; growth arrest and DNA damage inducible, beta = GADD45B; growth arrest and DNA damage inducible, betaGADD45B; Cyclin A1 = CCNA1; Cyclin-dependent kinase-like 2 (CDC2-related kinase) = CDKL2; Cyclin A2 = CCNA2; exosome component 9: cyclin A DNA = EXOSC9; kelch/ankyrin repeat containing cyclin A1 interacting protein = KARCA1; cDNA_clone AA608568 890 CCNA2. Cases of repeated genes on the right represent different probes targeting the same gene.

To evaluate the effect of resistance to the O-6 methylating drugs BCNU and Temozolomide on CDC2 interacting proteins, the data set published by Bredel and coworkers was interrogated [16]. In these experiments, glioblastoma cell lines from three patients (initialed DI, LX, and ME) were derived before and after treatment. Treatment included surgical debulking, radiotherapy, and chemotherapy. The cell lines were then selected for resistance to the O-6 methylating drugs Temozolomide (TMZ) and BCNU, and gene expression analysis was performed (for further experimental details, see Bredel et al. [16]). Selected CDC2 interacting and G2 → M transitioning proteins were evaluated, and the log base 2 relative expression value of these genes was used to draw the heat map illustrated in Figure 7. The *Y*-axis of the heat map shows the genes, labeled on the right, and the hierarchical clustering dendrogram is shown on the left. No hierarchical clustering was performed for the treatment samples. Of note, cyclin B2 (CCNB2), CDC2, PLK1, and CKS2 all clustered together and were downregulated in cell lines that had become resistant to BCNU and TMZ in patients DI, LX, and ME, albeit to differing degrees. Also of interest was the upregulation of Cyclin A1 noted in patient LX with TMZ treatment. These data demonstrate that levels of CDC2 interacting proteins and G2 → M transition regulators are changed after treatment with BCNU and TMZ.

### 3.4. CDC2 Copy Number Affects Patient Survival in Astrocytic Neoplasms but Not Oligodendroglial Neoplasms

**Figure 8:**
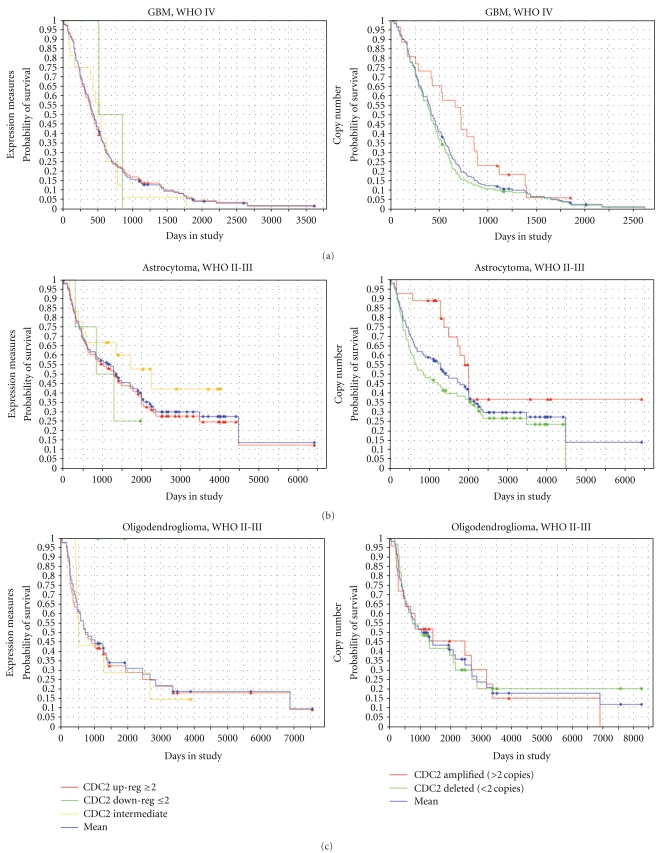
Kaplan-Meir survival curves for CDC2 expression levels and copy number in GBM, astrocytoma, and oligodendroglioma. Survival curves in GBM, astrocytoma grades II-III, and oligodendroglioma grades II-III with CDC2 expression measures (left column) and CDC2 copy number (right column). *X*-axis = time, *Y*-axis = survival probability. Up-reg = upregulation, Down-reg = downregulation. Increased CDC2 copy number is associated with increased survival in GBM and astrocytoma groups but not the oligodendroglioma group.

Select genes that interact with CDC2 (Kyoto Encyclopedia of Genes and Genomes (KEGG) pathways hsa04110 and hsa04115) were then queried in the REMBRANDT database. The results extracted from the database included Kaplan-Meyer survival curves for expression levels and copy number aberrations of CDC2, GSK3*β*, WEE1, CDK7/CAK, Cyclin A2, and Cyclin B1. Figure 8 demonstrates the Kaplan-Meyer survival curves in GBM, astrocytoma WHO grades II-III, and oligodendroglioma WHO grades II-III for CDC2 gene expression and CDC2 copy number. Data on ependymomas was not available. Numerical data associated with these graphs are presented in Table 2. In the GBM and the astrocytoma group, CDC2 gene amplification was associated with increased survival (log ranked *P-*value = .029 for each group), whereas CDC2 amplification did not significantly lead to increased survival in the oligodendroglioma group (log ranked *P-*value = .86). 

**Figure 9:**
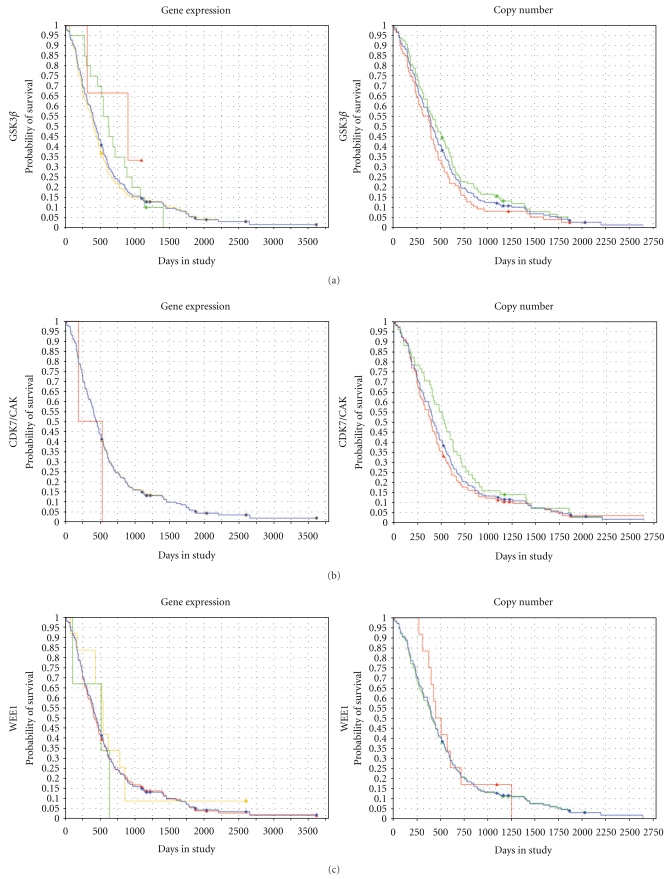

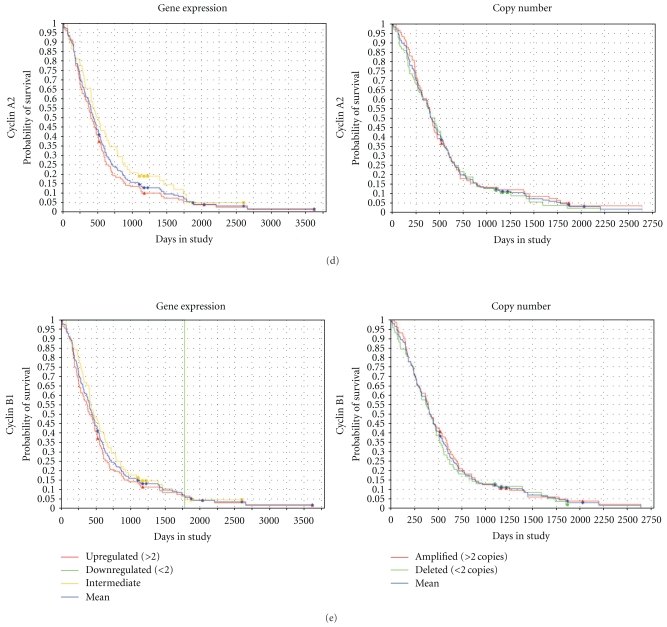
Kaplan-Meir survival curves for GSK3*β*, CDK7/CAK, WEE1, Cyclin A2, and Cyclin B1 in GBM. The molecule queried in the REMBRANDT database is listed on the left hand side of the panels. These molecules are known to interact with CDC2. Left column is the gene expression curve; right column is the copy number curve. No significant difference is noted in survival with gene expression or copy number (log ranked *P*-value >.05 in all instances). *X*-axis of each graph is time, *Y*-axis of each graph is survival probability.

**Figure 10:**
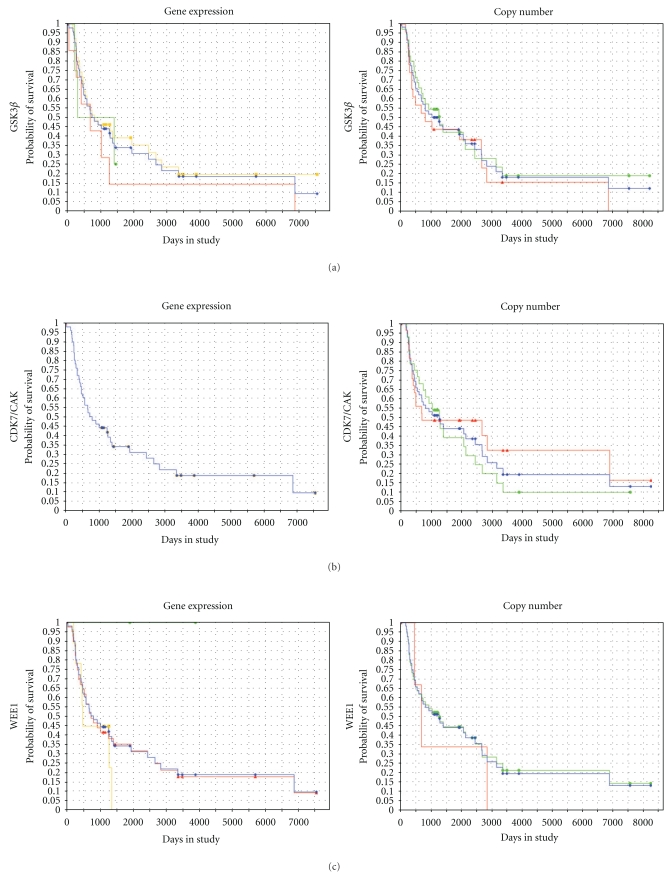

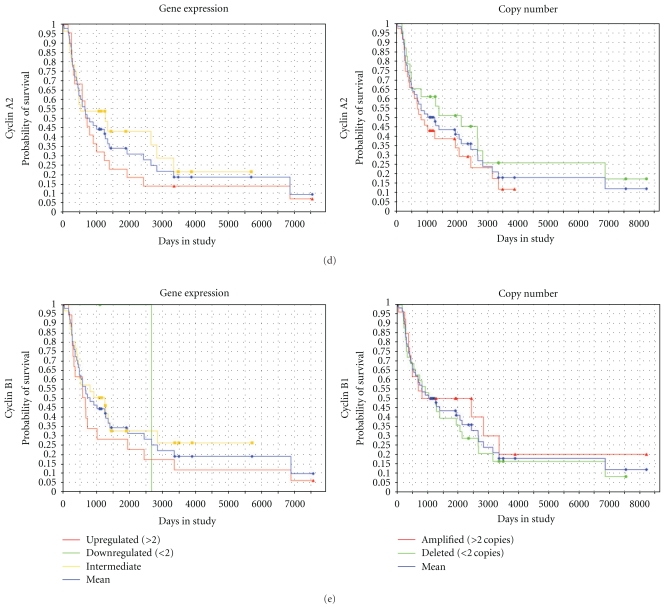
Kaplan-Meir survival curves for GSK3*β*, CDK7/CAK, WEE1, Cyclin A2, and Cyclin B1 in oligodendroglioma, WHO grades II-III. The molecule queried in the REMBRANDT database is listed on the left hand side of the panels. These molecules are known to interact with CDC2. Left column is the gene expression curve; right column is the copy number curve. No significant difference is noted in survival with gene expression or copy number (log ranked *P*-value >.05 in all instances). *X*-axis of each graph is time, *Y*-axis of each graph is survival probability.

To evaluate if CDC2-interacting proteins showed similar effects on patient survival, similar queries for GSK3*β*, WEE1, CDK7/CAK, Cyclin A2, and Cyclin B1 were performed. The results of these queries are illustrated in Figure 9 (GBM) and Figure 10 (oligodendroglioma WHO grades II-III), while the numerical data are presented in Table 3 (GBM) and Table 4 (oligodendroglioma WHO grades II-III). No significant difference, as determined by log ranked *P*-value, was seen on patient survival with expression or copy number changes in any of the involved genes. Overall, the trends of expression (i.e., upregulation versus downregulation versus intermediate expression) were very similar between the GBM and oligodendroglioma groups. However, Cyclin A2 was more commonly amplified in the oligodendroglioma group relative the GBM group (Pearson's Chi-squared test with Yate's continuity, *X* ^2^ = 12.5, *df* = 1, *P*-value = .00004).

## 4. Discussion

Many investigators have reported studies in which either pharmacological or radiological induced cell cycle arrest at the G2 → M checkpoint slows GBM growth [3, 5, 18–22]. These data are in overall concurrence with the notion that increased CDC2 copy number is associated with increased survival in astrocytic neoplasms and underscore the importance of the G2 → M cell cycle checkpoint in GBM. However, similar results in oligodendroglioma and ependymoma have not been reported. We found no studies in the literature that examined the CDC2 or GSK3*β* activation states in these tumors. GBM's showed higher labeling of both the promitotic pThr161CDC2 and antimitotic pTyr15CDC2 relative to both oligodendroglioma and ependymoma. In addition, promitotic CDC2 levels (pThr161) positively correlated with the histologic grade in oligodendroglioma. This is of no surprise, as similar findings with other mitotic markers have been reported in glioma [14, 23, 24]. Similarly, all ependymomas with elevated pThr161CDC2 levels occurred in pediatric patients, possibly reflecting the reportedly more proliferative behavior of these neoplasms in this age group [25, 26]. The positive correlation between oligodendroglioma grade and phospho-CDC2 raises the possibility that phospho-CDC2 labeling may correlate with tumor behavior. 

Tyrosine-15 phosphorylated CDC2 is expected to prevent cells to enter mitosis, whereas threonine-161 phosphorylation promotes mitosis. 15 of 20 WHO grade II oligodendrogliomas, 16 of 20 ependymomas, and 5 of 17 of the WHO grade III oligodendrogliomas showed low pTyr15CDC2 with low pThr161CDC2 labeling. However, dual elevation of pTyr15- and pThr161CDC2 was seen nearly in half of GBM patients. Elevations of both pTyr15- and pThr161-CDC2 are unexpected and is of uncertain significance. Although we have found no reports that suggest phosphorylation at both residues, Tyr15 and Thr161 are mutually exclusive, it is nevertheless unclear what function a dual phosphorylated CDC2 molecule would have. Alternatively, two intracellular pools of CDC2 may be present, with each pool diverted towards one phosphorylation state. In this scenario, the intracellular ratio of pTyr15CDC2 to pThr161CDC2 would determine the final fate of the cell. As the CDC2 phospho-specific antibodies used are both derived from immunized rabbits, we were unable to perform dual immunolabeling to determine if the same cells with elevated pTyr15CDC2 also were pThr161CDC2 positive, and hence we cannot exclude that the elevated labeling of these two epitopes are present in different cells of the same tumor. 

Hirose et al. showed that temozolomide (TMZ) treatment of glioma cell lines *in vitro *leads to CDC2 phosphorylation at Tyr15, and that inhibition of cell cycle progression by pTyr15CDC2 may be an important step in TMZ-induced cytotoxicity [20]. Suppression of CDC2's promitotic activity has been reported in many other chemotherapeutic agents [18, 27–30]. At least a subset of cells in GBM and anaplastic oligodendroglioma is capable of proceeding with mitosis despite signals that ought to prevent such an act (i.e., phosphorylation at Tyr15CDC2, see Figure 4). In addition, GBM cell lines selected *in vitro* for TMZ resistance showed downregulations in CDC2. This raises the question of whether or not a glioma cell, which is capable of proceeding to mitosis despite the presence of an arrest signal (i.e., pTyr15CDC2), would be responsive to drugs that require a G2 → M arrest. Additional prospective studies of basal pTyr15CDC2 levels in GBM and their response to TMZ treatment are thus necessary to evaluate the use of pTyr15CDC2 labeling as a prognostic indicator of survival in a manner similar to CDC2 copy number.

The distinct subcellular localizations noted for CDC2 and GSK3*β* in the different tumor types is interesting and may suggest distinct biological functions. Studies performed using antibodies that detect total CDC2 show that its subcellular localization changes in relation to cell cycle in yeast, plants, and animals [31–33]. CDC2 is located in the nucleus where it is tightly bound to chromatin [31]. During mitosis, CDC2 localizes to the centrosomes, where it closely interacts with spindle-associated microtubules, regulating their stability through phosphorylation [34–37]. The presence of activated CDC2 (pThr151CDC2) in the cytoplasm in GBM cells may thus suggest a role in microtubular reorganization in those cells. Of interest to the nuclear GSK3*β* noted in GBM and one case of anaplastic oligodendroglima is a report by Caspi et al. showing that nuclear GSK3*β* actively inhibits the canonical Wnt/*β*-catenin signaling pathway through a nonphosphorylation-dependent mechanism [38]. In summary, the differences in subcellular localization of CDC2 and GSK3*β* noted in GBM and anaplastic oligodendroglioma suggests that in those cells these with distinct subcellular localization, these molecules may have unique functions.

## 5. Conclusions

GBM showed elevated levels of activated (pThr161CDC2) and inhibited (Tyr15CDC2) relative to oligodendroglioma and ependymoma. This suggests that G2  →  M transition regulation is highly important in GBM. In support of this, glioma cell lines show changes in gene expression in CDC2 interacting genes after developing resistance to TMZ, a known target of the G2 → M phase transition. In addition, increased CDC2 copy number leads to increased patient survival in astrocytomas WHO II–IV, but not oligodendroglioma.

## Figures and Tables

**Figure 3:**
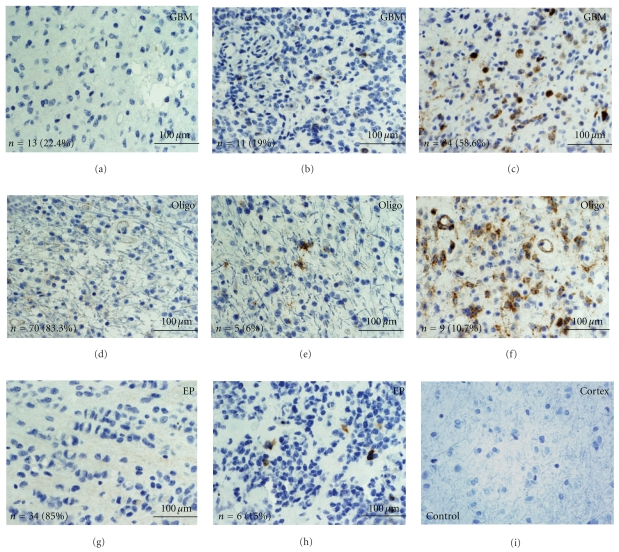
pTyr15CDC2 levels in glial tumors. Tissue arrays for GBM (a–c), oligodendroglioma (d–f), and ependymoma (g–h) were stained with anti-pTyr15CDC2 antibodies. Panel (i) demonstrates immunostaining in cortex of a control sample. Groups were separated into low labeling (a, d, and g), intermediate labeling (b, e, and h), and high labeling (c, and f) representative photographs are illustrated. Bottom left of each panel shows the total number of cores (*n*) and their proportion of total cores for the low, intermediate, and high labeling group. No cores of ependymoma showed high pTyr15CDC2 labeling. (GBM = glioblastoma; Oligo = oligodendroglioma; EP = ependymoma; scale bar in yellow represents 100 *μ*m).

**Table 1:** Immunohistochemical labeling profiles in glioma. Immunohistochemical labeling for pThr161CDC2, pTyr15CDC2, pSer9GSK3*β* and EGFR are represented in each glioma cohort. Numbers represent total number of patients with high (H), intermediate (I), or low (L) labeling indexes.

	Mean age	n	pThr161CDC2			pTyr15CDC2			pSer9GSK3*β*			EGFR		
			H	L	I	H	L	I	H	L	I	H	L	I
Glioblastoma, WHO IV	53.6	45	31	4	10	26	7	12	20	14	11	5	11	29

Anaplastic Oligodenroglioma, WHO III	46	17	8	3	6	4	3	8	10	2	5	0	1	16

Oligodendroglioma, WHO II	38.5	20	2	3	15	2	1	17	7	6	7	0	2	19

Ependymoma, WHO II (Pediatric)	6.2	9	2	1	6	0	2	6	1	2	6	0	0	9

Ependymoma, WHO II (Adult)	37.8	11	0	0	11	0	1	10	1	3	7	0	0	11

**Table:** (a)

Statistic	GBM,WHO IV	Astrocytoma,WHO II-III	OligodendrogliomaWHO II-III
Upregulated expression (*n*)	163	83	41
Downregulated expression (*n*)	2	4	2
Intermediate expression (*n*)	16	18	7
Up versus down *P*-value	.78	.72	.16
Up versus Int *P*-value	.77	.23	.99
Down versus Int *P*-value	.70	.39	.17
Up versus other *P*-value	.86	.34	.52
Down versus other *P*-value	.78	.64	.16
Int versus other *P*-value	.76	.22	.89

**Table:** (b)

Tumor	Number deleted (<2*x*)	Number amplified (>2*x*)	Log-rank *P*-value
GBM, WHO IV	160	26	.029
Astrocytoma, WHO II-III	75	27	.029
Oligodendroglioma, WHO II-III	33	25	.86

**Table:** (a)

Statistic	GSK3*β*	CDK7/CAK	WEE1	Cyclin A2	Cyclin B1
Upregulated expression (*n*)	3	2	166	123	111
Downregulated expression (*n*)	20	0	3	0	1
Intermediate expression (*n*)	158	179	12	58	69
Up versus down *P*-value	.46	n/a	.61	n/a	.28
Up versus Int *P*-value	.27	.42	.47	.11	.26
Down versus Int *P*-value	.26	n/a	.36	n/a	.28
Up versus other *P*-value	.29	n/a	.64	n/a	.21
Down versus other *P*-value	.30	n/a	.58	n/a	.28
Int versus other *P*-value	.16	n/a	.46	n/a	.32

**Table:** (b)

Statistic	GSK3*β*	CDK7/CAK	WEE1	Cyclin A2	Cyclin B1
Number deleted (<2*x*)	87	130	12	88	108
Number amplified (>2*x*)	99	51	169	98	78
*P*-value	.09	.14	.62	.65	.54

**Table:** (a)

Statistic	GSK3*β*	CDK7/CAK	WEE1	Cyclin A2	Cyclin B1
Upregulated expression (*n*)	7	0	39	22	18
Downregulated expression (*n*)	4	0	2	0	2
Intermediate expression (*n*)	39	50	9	28	30
Up versus down *P*-value	.64	n/a	.10	n/a	.27
Up versus Int *P*-value	.3	n/a	.48	.31	.29
Down versus Int *P*-value	.60	n/a	.047	n/a	.54
Up versus other *P*-value	.32	n/a	.76	n/a	.23
Down versus other *P*-value	.73	n/a	.086	n/a	.41
Int versus other *P*-value	.29	n/a	.34	n/a	.44

**Table:** (b)

Statistic	GSK3*β*	CDK7/CAK	WEE1	Cyclin A2	Cyclin B1
Number deleted (<2*x*)	23	27	3	11	36
Number amplified (>2*x*)	35	28	52	45	32
*P*-value	.47	.54	.57	.27	.50
